# Associations of early-pregnancy triiodothyronine and triglycerides with hypertensive disorders of pregnancy: a retrospective cohort study

**DOI:** 10.3389/fendo.2026.1975129

**Published:** 2026-10-09

**Authors:** Yanan Li, Jiabin Ren, Shuai Yang, Yulai Zhou, Zixuan Huang, Sichen Lv, Weibin Wu, Jianxia Fan

**Affiliations:** 1 The International Peace Maternity and Child Health Hospital, School of Medicine, Shanghai Jiao Tong University, Shanghai, China; 2 Shanghai Key Laboratory of Embryo Original Diseases, Shanghai, China

**Keywords:** hypertensive disorders of pregnancy, mediation analysis, preeclampsia, triglycerides, triiodothyronine

## Abstract

**Background:**

Thyroid hormones are associated with hypertensive disorders of pregnancy (HDP), but the contribution of triiodothyronine (T3), the biologically active thyroid hormone, remains insufficiently understood. Given the established role of T3 in lipid homeostasis and the association of triglycerides (TG) with HDP, we aimed to examine the associations of early-pregnancy T3 and TG levels with HDP and evaluate the potential role of TG in the association between T3 and HDP.

**Methods:**

This retrospective cohort study included 13,915 pregnant women who underwent prenatal screening between January 2020 and December 2021. Multivariable logistic regression was used to examine the associations of early-pregnancy free triiodothyronine (FT3), total triiodothyronine (TT3), and TG with HDP and its subtypes, while linear regression was used to assess the associations of FT3 and TT3 with TG. Mediation analysis assessed statistical indirect associations through TG, and multiplicative interaction analysis evaluated interactions between T3 and TG.

**Results:**

Higher early-pregnancy FT3, TT3, and TG levels were associated with higher odds of HDP (all *P* < 0.05), and both FT3 and TT3 were positively associated with TG (*P* < 0.05). Mediation analysis identified significant indirect associations through TG, representing an estimated 34.1% and 33.5% of the observed associations of FT3 and TT3 with HDP, respectively. Comparable indirect associations were observed for preeclampsia (PE), with estimated proportions of 18.6% and 28.3%, respectively. For gestational hypertension (GH), an indirect association through TG was observed for TT3 (estimated proportion, 42.0%), whereas the corresponding proportion for FT3 was not interpreted because the total association was not statistically significant. No evidence of multiplicative interaction between T3 and TG was observed in relation to HDP.

**Conclusion:**

Higher early-pregnancy FT3 and TT3 levels were associated with higher odds of HDP, particularly PE. TG may represent a potential metabolic component involved in the observed associations between T3 and HDP.

## Introduction

Hypertensive disorders of pregnancy (HDP) are among the most common pregnancy complications and remain leading causes of maternal morbidity and mortality worldwide (1, 2). Previous studies have shown that hypothyroidism, subclinical hypothyroidism (SCH), and isolated maternal hypothyroxinemia (IMH) are associated with higher odds of HDP (3–8). Most studies have focused on free thyroxine (FT4) and thyroid-stimulating hormone (TSH), whereas the association between triiodothyronine (T3), the biologically active thyroid hormone, and HDP remains insufficiently understood. Experimental studies have also suggested that altered T3 exposure may impair trophoblast proliferation and invasion, processes that are important for normal placental development and are implicated in the pathogenesis of HDP (9, 10). These findings support further investigation of the association between early-pregnancy T3 levels and subsequent HDP.

Thyroid hormones, particularly T3, regulate lipid metabolism through pathways involved in hepatic lipogenesis, fatty-acid β-oxidation, and lipoprotein metabolism (11–13). Consistent with this regulatory role, levothyroxine treatment has been reported to improve lipid profiles in both pregnant and nonpregnant women with subclinical hypothyroidism (14, 15). Meanwhile, prospective studies and meta-analyses show that higher levels of triglycerides (TG), low-density lipoprotein (LDL) cholesterol, and non-high-density lipoprotein cholesterol are associated with subsequent HDP (16–18). TG-rich lipoproteins and released fatty acids may contribute to oxidative stress, inflammation, and endothelial dysfunction, processes central to preeclampsia (PE) (9, 19, 20). Collectively, these biological and epidemiological findings raise the possibility that lipid metabolism may represent a potential metabolic context involved in the relationship between thyroid hormone status and HDP. However, the complex relationships among early-pregnancy T3 levels, circulating lipid profiles, particularly TG, and subsequent HDP remain insufficiently understood.

Accordingly, this study aimed to examine the associations of early-pregnancy T3 levels with subsequent HDP and to explore the potential role of TG in these associations.

## Methods

### Study population

The study protocol received approval from the Medical Ethics Committee of the International Peace Maternity and Child Health Hospital (IPMCH), School of Medicine, Shanghai Jiao Tong University (Approval No. GKLW 2016-69). Written informed consent was obtained from all participants.

This retrospective cohort study included 19,141 pregnant women who underwent prenatal screening at IPMCH between January 2020 and December 2021. The inclusion criteria were: 1) singleton pregnancy; 2) Han Chinese ethnicity; and 3) residence in an iodine-sufficient area. Women were excluded if they met any of the following criteria: 1) pregestational hypertension, thyroid disease, hyperlipidemia, diabetes mellitus; 2) a history of hypertensive disorders of pregnancy; or 3) thyroid and lipid biomarker measurements unavailable within 9-13 weeks of gestation. A total of 13,915 pregnant women were ultimately included in the final analysis (**Figure 1**).

**Figure 1:**
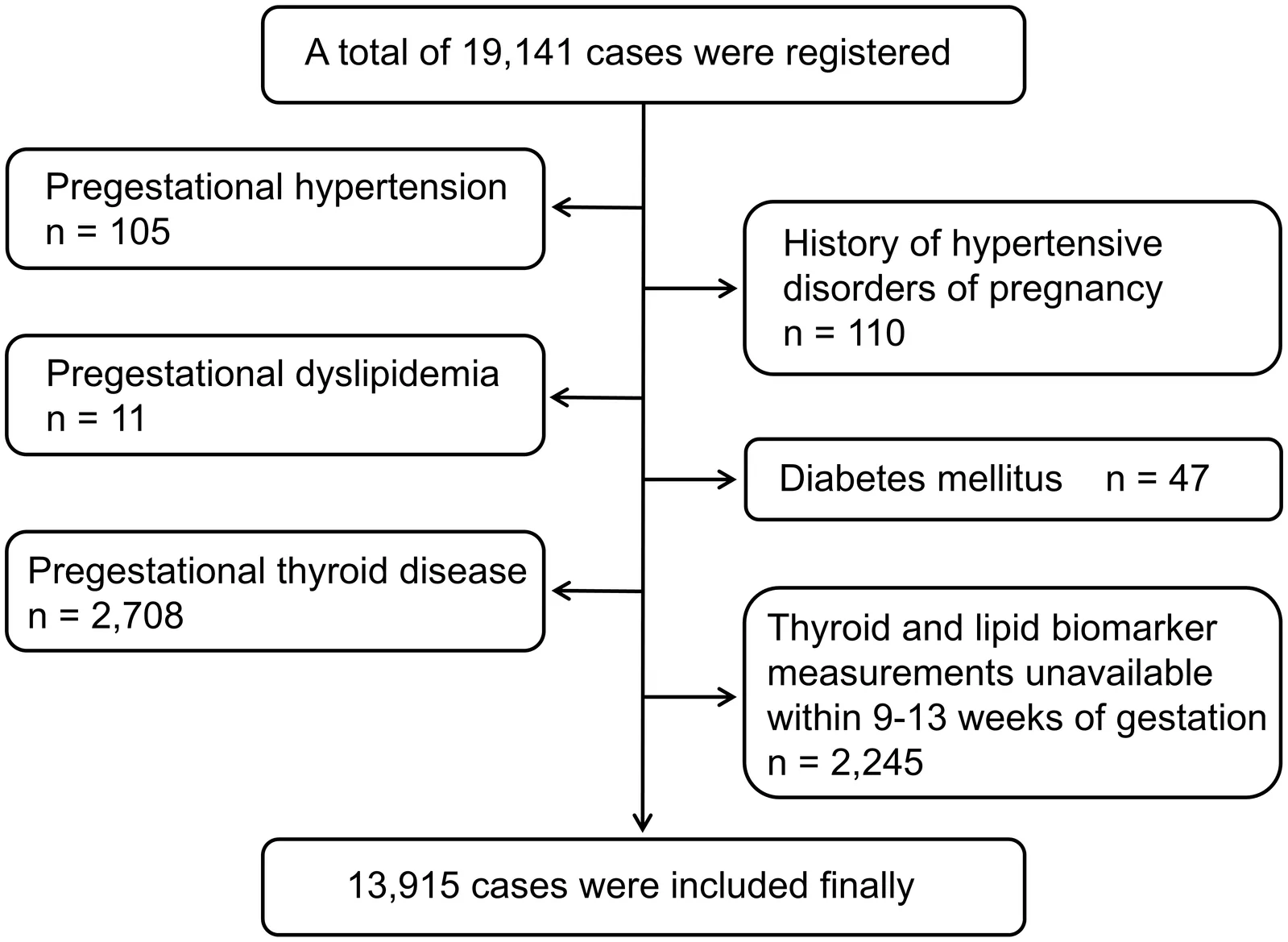
Flowchart of the study.

### Data collection

At the initial visit, maternal demographic and clinical information, including age, parity, last menstrual period (LMP), mode of conception, educational level, and medical history, was obtained through structured interviews. Pre-pregnancy body mass index (BMI) was calculated using self-reported pre-pregnancy weight and height, which was measured by trained nursing staff during early pregnancy. Women with a BMI of ≥24.0 or <24.0 kg/m^2^ were defined as overweight and obese (OWO) and non-OWO, respectively (21). Gestational age was initially estimated based on the reported LMP and subsequently confirmed or adjusted through first-trimester ultrasound examination. Fasting blood samples were obtained from pregnant women during early pregnancy (9-13 weeks of gestation). Blood was drawn from the median cubital vein, centrifuged, and the serum was separated within six hours of collection. The concentrations of free triiodothyronine (FT3), total triiodothyronine (TT3), FT4, total thyroxine (TT4), TSH, thyroid peroxidase antibodies (TPOAb), and thyroglobulin antibodies (TgAb) were quantified using the Architect i2000 immunoassay system (Abbott, Chicago, IL, USA), following the manufacturer’s standardized protocol. A TPOAb concentration exceeding 5.61 IU/mL is considered positive, as is a TgAb concentration exceeding 4.11 IU/mL, according to manufacturer defined cut-offs. Lipid profiles, including serum TG, total cholesterol (TC), high-density lipoprotein (HDL), LDL, apolipoprotein A (Apo A), apolipoprotein B (Apo B), and lipoprotein(a) (Lp(a)) were measured using commercial enzymatic colorimetric assays (Roche Diagnostics, Mannheim, Germany). These assays were processed using a Cobas c702 analyzer, with intra-assay and inter-assay coefficients of variation maintained at <5%.

### Diagnostic criteria

HDP was defined as either gestational hypertension (GH) or PE. GH was defined as *de novo* systolic blood pressure of ≥140 mmHg and/or diastolic blood pressure of ≥90 mmHg after 20 weeks of gestation, confirmed by a second measurement at least 4 hours later, in the absence of proteinuria or other features of PE. PE was defined as hypertension accompanied by proteinuria or at least one new-onset feature of maternal organ dysfunction. Proteinuria was defined as a spot urine protein-to-creatinine ratio of ≥0.3 mg/mg, urinary protein excretion of ≥0.3 g in a 24-hour urine collection, or a urine dipstick result of ≥1+ when quantitative testing was unavailable. Other qualifying features of maternal organ dysfunction included neurological complications such as severe headaches; pulmonary oedema; renal impairment, defined as a serum creatinine concentration of ≥1 mg/dL; and liver involvement, indicated by elevated transaminase levels (1, 22).

### Statistical analysis

Continuous variables with approximately normal distributions are presented as mean ± standard deviation (SD) and were compared using the independent-samples t test. Skewed continuous variables are presented as median (first quartile [Q1], third quartile [Q3]) and were compared using the Mann-Whitney U test. Categorical variables are presented as numbers and percentages and were compared using the chi-square test or Fisher’s exact test, as appropriate. Multivariable logistic regression analyses were performed to examine the associations of early-pregnancy FT3, TT3, and TG with HDP, with results reported as odds ratios (ORs) and 95% confidence intervals (CIs). Linear regression analyses were used to assess the association of FT3 and TT3 with TG, with the results reported as β coefficients and 95% CIs. We applied restricted cubic splines utilizing three knots to assess potential nonlinearity.

Given the close relationships of T3 with lipid profiles and HDP, mediation analyses were conducted to examine whether the observed associations of early-pregnancy FT3 and TT3 with HDP included a statistical indirect component through lipid biomarkers. TG was prespecified as the primary mediator, whereas TC, HDL, LDL, Apo A, Apo B, and Lp(a) were examined as exploratory mediators. HDP was the primary outcome, while GH and PE were examined in secondary analysis of HDP subtypes. Linear regression was used for continuous mediators and logistic regression for binary outcomes. All mediation models were adjusted for maternal age, parity, pre-pregnancy BMI, mode of conception, educational level, family history of hypertension, and thyroid antibody positivity. The exposure contrast was defined as an increase from the mean to one standard deviation above the mean for FT3 and TT3, corresponding to increases of 0.94 pmol/L and 0.37 nmol/L, respectively. The total effect (TE), pure natural direct effect (PNDE), and total natural indirect effect (TNIE) were estimated on the OR scale using regression-based direct counterfactual imputation, with 95% percentile CIs and *P* values obtained by bootstrap resampling. The proportion mediated was interpreted only when the TE and TNIE were statistically significant, directionally concordant, and sufficiently stable. Additional exploratory analyses of the TG-related indirect associations were performed in women without pre-pregnancy overweight or obesity (OWO) and, separately, in women who did not develop gestational diabetes mellitus (GDM).

To account for multiple comparisons, Holm correction was applied across the two natural indirect-effect tests involving FT3 and TT3 for the primary HDP outcome and separately across the four corresponding tests for the secondary outcomes of GH and PE. For the exploratory lipid mediators, the Benjamini-Hochberg false discovery rate procedure was applied across the 36 natural indirect-effect tests involving six lipid biomarkers, two thyroid hormone exposures, and three hypertensive outcomes. Multiplicity adjustment was restricted to the TNIE, which represented the primary tests of mediation.

In addition, multiplicative interactions were evaluated by including T3 × TG product terms in multivariable logistic regression models.

All statistical tests were two-sided, and *P* < 0.05 was considered statistically significant. Statistical analyses were performed using IBM SPSS Statistics, version 26.0 (IBM Corp., Armonk, NY, USA), and R, version 4.3.3 (R Foundation for Statistical Computing, Vienna, Austria). Mediation analyses were conducted using the CMAverse package.

## Results

### Characteristics of the participants

**Table 1** summarized the clinical characteristics of the participants. Among the 13,915 pregnant women enrolled, 998 cases of HDP were identified, corresponding to a prevalence rate of 7.17%. Compared to the non-HDP group, women in the HDP group had a significantly higher pre-pregnancy BMI (22.71 ± 3.36 vs. 21.11 ± 2.71 kg/m², *P* < 0.001) and a higher prevalence of assisted reproductive technology (ART) use (15.0% vs. 8.9%, *P* < 0.001). Women with HDP were also more likely to be nulliparous. Moreover, a family history of hypertension was more common among women with HDP (27.3% vs. 18.6%, *P* < 0.001).

**Table 1:** Comparison of clinical characteristics between HDP and non-HDP pregnant women.

Characteristics	Total (n = 13,915)	HDP (n = 998)	Non-HDP (n = 12,917)	*P*
Age (years)	31.66 ± 3.86	31.71 ± 3.98	31.66 ± 3.85	0.639
Pre-BMI (kg/m^2^)	21.23 ± 2.79	22.71 ± 3.36	21.11 ± 2.71	< 0.001
Pre-BMI category (kg/m^2^)				< 0.001
< 18.5	1,916 (13.8%)	74 (7.4%)	1,842 (14.3%)	
18.5 - < 24	9,947 (71.5%)	595 (59.6%)	9,352 (72.4%)	
24 - < 28	1,709 (12.3%)	261 (26.2%)	1,448 (11.2%)	
≥ 28	343 (2.5%)	68 (6.8%)	275 (2.1%)	
Parity				< 0.001
Nulliparous	10,170 (73.1%)	861 (86.3%)	9,309 (72.1%)	
Multiparous	3,745 (26.9%)	137 (13.7%)	3,608 (27.9%)	
Mode of conception				< 0.001
Spontaneous conception	12,610 (90.6%)	848 (85.0%)	11,762 (91.1%)	
ART	1,305 (9.4%)	150 (15.0%)	1,155 (8.9%)	
Education level				0.002
High school or lower	872 (6.3%)	68 (6.8%)	804 (6.2%)	
Bachelor	9,551 (68.6%)	727 (72.8%)	8,824 (68.3%)	
Master or PhD	3,492 (25.1%)	203 (20.3%)	3,289 (25.5%)	
Family history of hypertension				< 0.001
Without	11,235 (80.7%)	726 (72.7%)	10,509 (81.4%)	
With	2,680 (19.3%)	272 (27.3%)	2,408 (18.6%)	
FT3 (pmol/L)	5.29 ± 0.94	5.39 ± 0.91	5.28 ± 0.94	< 0.001
FT4 (pmol/L)	13.03 ± 1.69	12.91 ± 1.63	13.04 ± 1.69	0.016
TT3 (nmol/L)	2.00 ± 0.37	2.07 ± 0.37	1.99 ± 0.37	< 0.001
TT4 (nmol/L)	137.29 ± 24.73	135.99 ± 24.22	137.39 ± 24.77	0.086
TSH (mIU/L)	1.04 (0.54, 1.66)	1.10 (0.57, 1.67)	1.03 (0.54, 1.66)	0.123
TPOAb (IU/mL)	0.40 (0.40, 0.80)	0.40 (0.40, 0.80)	0.40 (0.40, 0.80)	0.063
TPOAb (IU/mL)				0.361
< 5.61 (−)	12,673 (91.1%)	901 (90.3%)	11,772 (91.1%)	
≥ 5.61 (+)	1,242 (8.9%)	97 (9.7%)	1,145 (8.9%)	
TgAb (IU/mL)	0.90 (0.60, 1.80)	1.00 (0.60, 2.10)	0.90 (0.60, 1.80)	0.229
TgAb (IU/mL)				0.401
< 4.11 (−)	11,839 (85.1%)	840 (84.2%)	10,999 (85.2%)	
≥ 4.11 (+)	2,076 (14.9%)	158 (15.8%)	1,918 (14.8%)	
TG (mmol/L)	1.20 (0.96, 1.52)	1.29 (1.02, 1.67)	1.19 (0.95, 1.51)	< 0.001
TC (mmol/L)	4.38 ± 0.73	4.45 ± 0.74	4.38 ± 0.73	0.001
HDL (mmol/L)	1.88 ± 0.38	1.82 ± 0.40	1.91 ± 0.38	< 0.001
LDL (mmol/L)	2.38 ± 0.60	2.49 ± 0.63	2.37 ± 0.60	< 0.001
Apo A (g/L)	1.84 ± 0.32	1.81 ± 0.26	1.84 ± 0.32	0.005
Apo B (g/L)	0.77 ± 0.15	0.80 ± 0.16	0.77 ± 0.15	< 0.001
Lp(a) (mg/L)	111.00 (64.00, 231.00)	105.00 (60.00, 224.75)	112.00 (64.00, 232.00)	0.045

Data are presented as mean ± SD, median (Q1, Q3), or n (%), as appropriate.

Apo A, apolipoprotein A; Apo B, apolipoprotein B; ART, assisted reproductive technology; FT3, free triiodothyronine; FT4, free thyroxine; HDL, high-density lipoprotein; HDP, hypertensive disorders of pregnancy; LDL, low-density lipoprotein; Lp(a), lipoprotein(a); PhD, doctor of philosophy; Pre-BMI, pre-pregnancy body mass index; SD, standard deviation; TC, total cholesterol; TG, triglyceride; TgAb, thyroglobulin antibody; TPOAb, thyroid peroxidase antibody; TSH, thyroid-stimulating hormone; TT3, total triiodothyronine; TT4, total thyroxine.

Women with HDP had lower FT4 levels (12.91 ± 1.63 vs. 13.04 ± 1.69 pmol/L, *P* = 0.016), but higher FT3 (5.39 ± 0.91 vs. 5.28 ± 0.94 pmol/L, *P* < 0.001) and TT3 (2.07 ± 0.37 vs. 1.99 ± 0.37 nmol/L, *P* < 0.001) levels compared to those without HDP. Additionally, the HDP group had higher levels of TG (1.29 vs. 1.19 mmol/L, *P* < 0.001), TC (4.45 ± 0.74 vs. 4.38 ± 0.73 mmol/L, *P* = 0.001), LDL (2.49 ± 0.63 vs. 2.37 ± 0.60 mmol/L, *P* < 0.001), and Apo B (0.80 ± 0.16 vs. 0.77 ± 0.15 g/L, *P* < 0.001), while HDL (1.82 ± 0.40 vs. 1.91 ± 0.38 mmol/L, *P* < 0.001), Apo A (1.81 ± 0.26 vs. 1.84 ± 0.32 g/L, *P* = 0.005) and Lp(a) (105.00 vs. 112.00 mg/L, *P* = 0.045) were lower in the HDP group.

### Association of early-pregnancy T3 and TG levels with HDP

As shown in **Table 2** and **Figure 2**, levels of FT3, TT3, and TG in early pregnancy were positively associated with the risk of HDP (all *P* < 0.05). These associations remained statistically significant after adjusting for potential confounders including FT4, TSH, TPOAb positivity and TgAb positivity. Subsequent subgroup analyses were conducted to examine the association of these biomarkers with PE and GH separately. After adjusting for potential confounders, FT3 (OR, 1.17; 95% CI, 1.05-1.31; *P* = 0.006), TT3 (OR, 1.55; 95% CI, 1.18-2.03; *P* = 0.001) and TG (OR, 1.50; 95% CI, 1.27-1.77; *P* < 0.001) demonstrated a significant positive association with PE. TT3 and TG levels remained significantly associated with an increased risk of GH. However, the association between FT3 and GH was no longer significant after adjusting for age, parity, pre-pregnancy BMI, mode of conception, education level, family history of hypertension, FT4, TSH, TPOAb positivity and TgAb positivity. Furthermore, significant positive associations of FT3 and TT3 with TG levels were observed (all *P* < 0.001).

**Table 2:** Associations of early-pregnancy T3 and TG with HDP and its subtypes, and associations between T3 and TG.

Outcome	Exposure	Model 1	*P*	Model 2	*P*	Model 3	*P*
HDP	FT3	1.13 (1.06, 1.20)	< 0.001	1.08 (1.01, 1.16)	0.021	1.10 (1.03, 1.19)	0.009
	TT3	1.69 (1.44, 1.98)	< 0.001	1.42 (1.20, 1.69)	< 0.001	1.48 (1.23, 1.76)	< 0.001
	TG	1.62 (1.46, 1.80)	< 0.001	1.45 (1.29, 1.62)	< 0.001	1.45 (1.29, 1.63)	< 0.001
PE	FT3	1.18 (1.08, 1.30)	< 0.001	1.16 (1.05, 1.28)	0.004	1.17 (1.05, 1.31)	0.006
	TT3	1.74 (1.37, 2.22)	< 0.001	1.54 (1.19, 1.98)	< 0.001	1.55 (1.18, 2.03)	0.001
	TG	1.60 (1.37, 1.87)	< 0.001	1.49 (1.26, 1.76)	< 0.001	1.50 (1.27, 1.77)	< 0.001
GH	FT3	1.09 (1.00, 1.18)	0.043	1.03 (0.95, 1.13)	0.485	1.06 (0.96, 1.16)	0.238
	TT3	1.63 (1.33, 1.99)	< 0.001	1.33 (1.08, 1.65)	0.009	1.40 (1.12, 1.76)	0.003
	TG	1.64 (1.44, 1.87)	< 0.001	1.44 (1.25, 1.66)	< 0.001	1.44 (1.25, 1.66)	< 0.001
TG	FT3	0.08 (0.07, 0.09)	< 0.001	0.07 (0.06, 0.08)	< 0.001	0.11 (0.10, 0.12)	< 0.001
	TT3	0.36 (0.34, 0.39)	< 0.001	0.34 (0.32, 0.36)	< 0.001	0.40 (0.38, 0.42)	< 0.001

Model 1: unadjusted model.

Model 2: adjusted for age, parity, pre-pregnancy BMI, mode of conception, education level, family history of hypertension.

Model 3: adjusted further for FT4, TSH, TPOAb positivity and TgAb positivity.

BMI, body mass index; FT3, free triiodothyronine; FT4, free thyroxine; GH, gestational hypertension; HDP, hypertensive disorders of pregnancy; PE, preeclampsia; TG, triglyceride; TgAb, thyroglobulin antibody; TPOAb, thyroid peroxidase antibody; TSH, thyroid-stimulating hormone; T3, triiodothyronine; TT3, total triiodothyronine.

**Figure 2:**
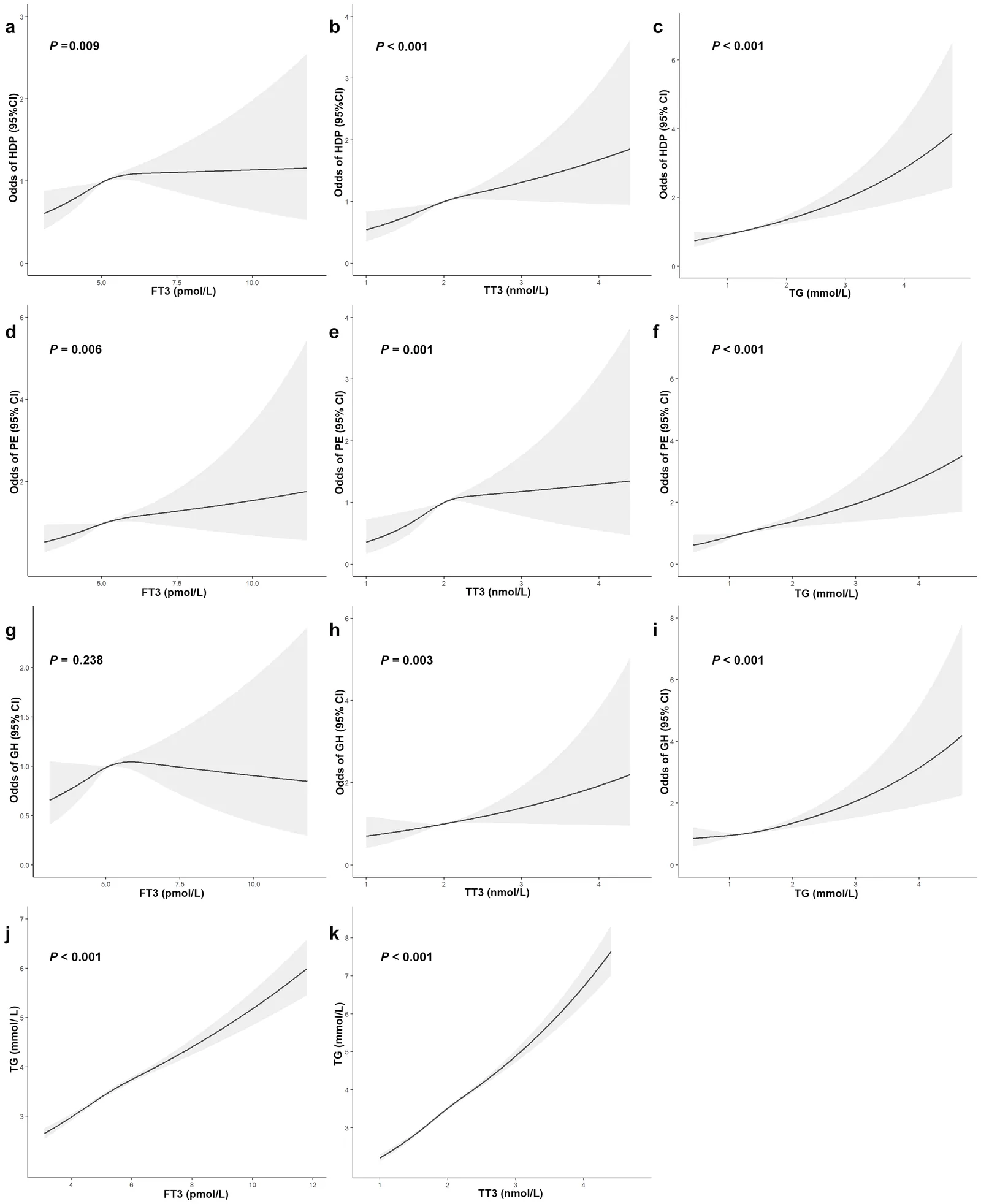
Associations of early-pregnancy T3 and TG with HDP and its subtypes, and associations between T3 and TG. **(a–c)** Associations of FT3, TT3, and TG with HDP, respectively; **(d–f)** associations of FT3, TT3, and TG with PE, respectively; **(g–i)** associations of FT3, TT3, and TG with GH, respectively; **(j, k)** associations of FT3 and TT3 with TG, respectively. FT3, free triiodothyronine; GH, gestational hypertension; HDP, hypertensive disorders of pregnancy; PE, preeclampsia; TG, triglyceride; T3, triiodothyronine; TT3, total triiodothyronine.

### Potential role of TG in the associations between T3 and HDP

Higher early-pregnancy FT3, TT3, and TG levels were associated with increased odds of HDP, and both FT3 and TT3 were positively associated with TG levels (**Table 2**). We further examined whether the observed T3-HDP associations included a statistical indirect component through TG. For FT3, the TE for HDP was 1.07 per 1-SD increase (0.94 pmol/L; 95% CI, 1.02-1.14; *P* = 0.008), comprising a PNDE of 1.05 (95% CI, 0.99-1.11; *P* = 0.100) and a TNIE through TG of 1.02 (95% CI, 1.02-1.03; *P* < 0.001). The indirect component represented an estimated 34.1% of the total association. For TT3, the TE and TNIE were 1.13 per 1-SD increase (0.37 nmol/L; 95% CI, 1.07-1.20; *P* < 0.001) and 1.04 (95% CI, 1.03-1.05, *P* < 0.001), respectively, with the indirect component accounting for an estimated 33.5% of the total association. In the analysis of HDP subtypes, statistically significant indirect associations through TG were observed for both FT3 and TT3 with PE, with estimated proportions of the total associations represented by the indirect components of 18.6% and 28.3%, respectively. For GH, the indirect component through TG was observed for TT3 (estimated proportion, 42.0%), the corresponding proportion for FT3 was not interpreted because the TE was not statistically significant. (**Table 3**; **Figure 3**). In addition, no evidence of multiplicative interaction between T3 and TG was observed for HDP risk (**Table 4**).

**Table 3:** Mediation analysis of early-pregnancy TG in the associations between T3 and HDP.

Outcome	Exposure	TE	*P*	PNDE	*P*	TNIE	*P*	*P*_adj_	Proportion mediated
HDP	FT3	1.07 (1.02, 1.14)	0.008	1.05 (0.99, 1.11)	0.100	1.02 (1.02, 1.03)	< 0.001	<0.001	34.1%
	TT3	1.13 (1.07, 1.20)	< 0.001	1.09 (1.03, 1.15)	< 0.001	1.04 (1.03, 1.05)	< 0.001	<0.001	33.5%
PE	FT3	1.14 (1.03, 1.24)	< 0.001	1.11 (1.01, 1.21)	0.040	1.02 (1.01, 1.04)	< 0.001	<0.001	18.6%
	TT3	1.17 (1.07, 1.27)	< 0.001	1.12 (1.02, 1.23)	0.012	1.04 (1.02, 1.06)	0.008	0.008	28.3%
GH	FT3	1.03 (0.95, 1.10)	0.556	1.00 (0.93, 1.08)	0.988	1.02 (1.01, 1.03)	< 0.001	<0.001	94.0%
	TT3	1.11 (1.03, 1.19)	0.008	1.06 (0.98, 1.15)	0.152	1.04 (1.02, 1.06)	< 0.001	<0.001	42.0%

Effect estimates correspond to a 1-SD increase in FT3 or TT3 (0.94 pmol/L and 0.37 nmol/L, respectively). Mediation analyses were adjusted for age, parity, pre-pregnancy BMI, mode of conception, education level, family history of hypertension, TPOAb positivity, and TgAb positivity.

BMI, body mass index; FT3, free triiodothyronine; GH, gestational hypertension; HDP, hypertensive disorders of pregnancy; PE, preeclampsia; PNDE, pure natural direct effect; SD, standard deviation; TE, total effect; TG, triglyceride; TgAb, thyroglobulin antibody; TNIE, total natural indirect effect; TPOAb, thyroid peroxidase antibody; T3, triiodothyronine; TT3, total triiodothyronine.

**Figure 3:**
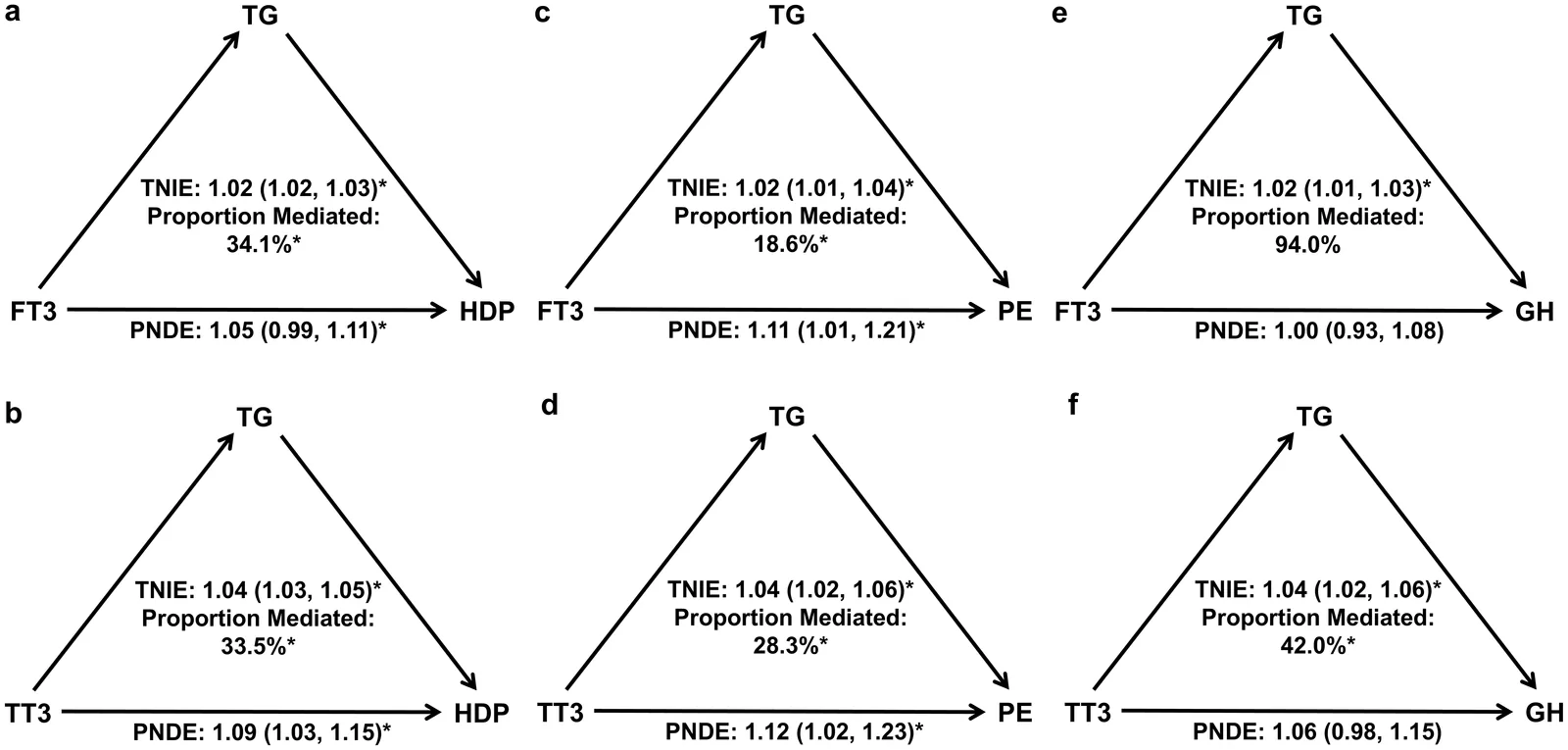
Directed acyclic graphs for analyses of the relationships among early-pregnancy T3, TG, and HDP and its subtypes. **(a, b)** Mediation analyses of FT3 and TT3, respectively, with HDP through TG; **(c, d)** mediation analyses of FT3 and TT3, respectively, with PE through TG; **(e, f)** mediation analyses of FT3 and TT3, respectively, with GH through TG. CI, confidence interval; FT3, free triiodothyronine; GH, gestational hypertension; HDP, hypertensive disorders of pregnancy; PE, preeclampsia; PNDE, pure natural direct effect; TG, triglyceride; TNIE, total natural indirect effect; T3, triiodothyronine; TT3, total triiodothyronine. *P < 0.05.

**Table 4:** Multiplicative interactions between early-pregnancy T3 and TG in relation to HDP.

Outcome	Interaction term	OR for interaction (95% CI)	*P* for interaction
**HDP**	**FT3×TG**	1.03 (0.93, 1.14)	0.571
	**TT3×TG**	0.98 (0.75, 1.29)	0.903

Analyses were adjusted for age, parity, pre-pregnancy BMI, mode of conception, education level, family history of hypertension, TPOAb positivity, and TgAb positivity.

BMI, body mass index; CIs, confidence intervals; FT3, free triiodothyronine; HDP, hypertensive disorders of pregnancy; ORs, odds ratios; TG, triglyceride; TgAb, thyroglobulin antibody; TPOAb, thyroid peroxidase antibody; TT3, total triiodothyronine; T3, triiodothyronine.

Among participants without OWO, similar indirect associations through TG were observed for both FT3 and TT3 with HDP, accounting for 27.9% and 28.1% of the respective total associations. The corresponding proportions were 16.6% and 23.1% for PE. For GH, the indirect association through TG accounted for 33.8% of the total association for TT3 (**Supplementary Table S1**). When women who subsequently developed GDM were excluded, similar indirect associations through TG were observed for both FT3 and TT3 with HDP, accounting for 27.0% and 24.4% of the respective total associations. The corresponding proportions were 14.9% and 19.2% for PE and 33.0% for the association between TT3 and GH (**Supplementary Table S2**).

### Exploratory mediation analysis of other lipid biomarkers in the associations between T3 and HDP

Further exploratory mediation analyses were conducted for other lipid biomarkers (**Supplementary Table S3**). After correction for multiple comparisons, only the indirect association between FT3 and HDP through HDL remained statistically significant (TNIE, 1.007; 95% CI, 1.003-1.012; adjusted *P* < 0.001), accounting for an estimated 9.9% of the total association. Several additional indirect associations were nominally significant but did not survive false discovery rate correction.

## Discussion

In this large retrospective cohort study, we observed that higher FT3, TT3 and TG levels in early pregnancy were associated with higher odds of HDP. For HDP subtypes, higher FT3, TT3, and TG levels were associated with PE, whereas TT3 and TG, but not FT3, remained significantly associated with GH after multivariable adjustment. Mediation analysis suggested statistical indirect associations through TG for both FT3 and TT3 in relation to HDP and PE, and for TT3 in relation to GH. Similar patterns were observed in exploratory analysis restricted to participants without OWO and, separately, to those without GDM. In exploratory analysis of other lipid biomarkers, only HDL showed an additional indirect association between FT3 and HDP after correction for multiple comparisons.

Previous studies of thyroid function and HDP have predominantly focused on FT4 and TSH, with hypothyroidism, SCH, and IMH consistently reported to be associated with higher odds of HDP (3–8). In contrast, the role of T3, the biologically active thyroid hormone, remains less well characterized. Our findings extend previous observations by showing that higher early-pregnancy FT3 and TT3 levels were associated with HDP and PE after adjustment for conventional thyroid-related factors including FT4, TSH, and thyroid antibody status. Several clinical studies have reported similar associations. Research found that women in the upper FT3 tertile had a higher incidence of PE and GH (18). A recent meta-analysis also reported a U-shaped association between FT3 and the risk of GH and PE, with higher TT3 levels associated with GH (23). Although the underlying mechanisms remain incompletely understood, several biological pathways may support the observed associations. T3 regulates multiple processes relevant to placental development, including trophoblast proliferation, invasion, angiogenesis, and inflammatory responses. Experimental studies have demonstrated that excessive T3 exposure may alter trophoblast behavior and disturb angiogenic factor balance, potentially affecting placental vascular development (24). T3 and epidermal growth factor (EGF) exhibited antiproliferative effects on an extravillous-like cell line (SGHPL-4). While EGF enhanced the survival of non-proliferative term primary cytotrophoblast cells and significantly enhanced invasion of fibrin gels by SGHPL-4 cells, these effects were mitigated by T3 (10). Given that impaired trophoblast invasion and abnormal placental angiogenesis are central features of HDP, particularly PE, altered T3 signaling may represent one biologically plausible pathway linking thyroid hormone status with pregnancy complications (9).

The relationship between maternal lipid abnormalities and HDP has been extensively investigated. Women who subsequently develop HDP often exhibit altered lipid profiles characterized by elevated TG, LDL cholesterol, and non-HDL cholesterol concentrations during pregnancy (16). Among these lipid parameters, elevated TG levels in early pregnancy have been consistently associated with subsequent development of PE and GH (16, 17, 25). Several biological mechanisms may explain these observations. Pregnancy requires substantial adaptation of lipid metabolism to support fetal growth; however, excessive accumulation of circulating TG may be associated with endothelial dysfunction, oxidative stress, and inflammatory activation (19). Moreover, free fatty acids and apolipoprotein-CIII released from TG promote oxidative stress and a pro-atherogenic response in macrophages and endothelial cells (20), which resulted in arterial vasospasm and disrupted vascular remodeling of the vessels. These processes collectively contributed to abnormal placentation and placental insufficiency (26), ultimately fostering the development of HDP.

Beyond the independent associations of T3 and TG with HDP, our findings suggest that alterations in lipid metabolism may represent a potential metabolic context associated with both T3 levels and HDP. Previous studies have established an important role of thyroid hormone signaling in lipid homeostasis, with T3 acting as a key regulator of hepatic lipid metabolism (27, 28). The liver is a major target organ of thyroid hormone action and expresses thyroid hormone receptors (THRs), particularly thyroid hormone receptor β (THRB), which plays a central role in hepatic lipid regulation (29). Upon binding to T3, THRB modulates downstream transcriptional networks involved in cholesterol transport, fatty-acid oxidation, mitochondrial function, lipoprotein clearance, and *de novo* lipogenesis (12), with key metabolic regulators such as sterol regulatory element-binding proteins (SREBPs), peroxisome proliferator-activated receptors (PPARs), and fibroblast growth factor 21 (FGF21) contributing to lipid homeostasis (30). Consistent with this possibility, previous clinical studies have reported positive associations between FT3 concentrations or FT3/FT4 ratios and adverse metabolic profiles, including dyslipidemia and gestational metabolic abnormalities (31–33). Higher FT3 levels and increased FT3/FT4 ratios have also been observed among women with GDM and have been associated with altered lipid and glucose metabolism (34–37). These findings support the possibility that altered T3-related metabolic regulation, particularly through lipid metabolism, may represent one potential biological context underlying the observed associations among T3, TG, and HDP. Further studies integrating longitudinal assessments of thyroid hormones, lipid profiles, and placental function are warranted to elucidate the biological mechanisms underlying these associations.

Several limitations should be considered. First, because T3 and TG were measured contemporaneously during early pregnancy, their temporal sequence could not be established. Accordingly, the mediation findings should be interpreted as reflecting statistical indirect associations through TG rather than confirming a causal T3–TG–HDP pathway. The possibility of shared metabolic determinants influencing both T3 and TG cannot be excluded, nor can reverse directionality, whereby TG may influence T3. Second, the retrospective design and the exclusion of women without available thyroid or lipid measurements within the prespecified early-pregnancy window may have introduced selection bias. Third, although we adjusted for a range of potential confounding factors, residual confounding from unmeasured or incompletely measured factors cannot be excluded. Finally, as this was a single-center study of Han Chinese women from an iodine-sufficient area, the generalizability of our findings may be limited. Future longitudinal, multicenter studies with repeated measurements of thyroid hormones and lipid profiles in more diverse populations are warranted to clarify temporal relationships and assess the generalizability of these findings.

## Conclusion

Our findings indicate that higher early-pregnancy FT3 and TT3 levels were associated with higher odds of HDP, particularly PE. Additionally, TG may represent a potential metabolic correlate in the observed associations between T3 and HDP. These findings provide further evidence of an interrelationship among thyroid hormone status, lipid metabolism, and HDP. Further prospective and mechanistic studies are warranted to elucidate the biological mechanisms underlying these associations.

## Data Availability

The raw data supporting the conclusions of this article will be made available by the authors, without undue reservation.

## Supplementary material

Supplementary Table S1 Mediation analysis of early-pregnancy TG in the relationships between T3 and HDP in non-OWO pregnant women. Effect estimates correspond to a 1-SD increase in FT3 or TT3 (0.94 pmol/L and 0.37 nmol/L, respectively). Mediation analyses were adjusted for age, parity, pre-pregnancy BMI, mode of conception, education level, family history of hypertension, TPOAb positivity, and TgAb positivity. BMI, body mass index; FT3, free triiodothyronine; GH, gestational hypertension; HDP, hypertensive disorders of pregnancy; OWO, overweight or obesity; PE, preeclampsia; PNDE, pure natural direct effect; SD, standard deviation; TE, total effect; TG, triglyceride; TNIE, total natural indirect effect; TgAb, thyroglobulin antibody; TPOAb, thyroid peroxidase antibody; T3, triiodothyronine; TT3, total triiodothyronine.

Supplementary Table S2 Mediation analysis of early-pregnancy TG in the relationships between T3 and HDP in non-GDM pregnant women. Effect estimates correspond to a 1-SD increase in FT3 or TT3 (0.94 pmol/L and 0.37 nmol/L, respectively). Mediation analyses were adjusted for age, parity, pre-pregnancy BMI, mode of conception, education level, family history of hypertension, TPOAb positivity, and TgAb positivity. BMI, body mass index; FT3, free triiodothyronine; GDM, gestational diabetes mellitus; GH, gestational hypertension; HDP, hypertensive disorders of pregnancy; PE, preeclampsia; PNDE, pure natural direct effect; SD, standard deviation; TE, total effect; TG, triglyceride; TNIE, total natural indirect effect; TgAb, thyroglobulin antibody; TPOAb, thyroid peroxidase antibody; T3, triiodothyronine; TT3, total triiodothyronine.

Supplementary Table S3 Exploratory mediation analysis of other lipid biomarkers in the associations between T3 and HDP. Effect estimates correspond to a 1-SD increase in FT3 or TT3 (0.94 pmol/L and 0.37 nmol/L, respectively). Mediation analyses were adjusted for age, parity, pre-pregnancy BMI, mode of conception, education level, family history of hypertension, TPOAb positivity, and TgAb positivity. Apo A, apolipoprotein A; Apo B, apolipoprotein B; BMI, body mass index; FT3, free triiodothyronine; GH, gestational hypertension; HDL, high-density lipoprotein; HDP, hypertensive disorders of pregnancy; LDL, low-density lipoprotein; Lp(a), lipoprotein(a); PE, preeclampsia; PNDE, pure natural direct effect; SD, standard deviation; TC, total cholesterol; TE, total effect; TG, triglyceride; TNIE, total natural indirect effect; TgAb, thyroglobulin antibody; TPOAb, thyroid peroxidase antibody; T3, triiodothyronine; TT3, total triiodothyronine.
