# Supplementary material for: Associations of early-pregnancy triiodothyronine and triglycerides with hypertensive disorders of pregnancy: a retrospective cohort study
Source: Front Endocrinol (Lausanne). 2026 Oct 9;17:1975129. doi: 10.3389/fendo.2026.1975129 (PMC13690663; doi:10.3389/fendo.2026.1975129)
Supplement: Supplementary file 1 [file Table1.doc]

**Table S1 Mediation analysis of early-pregnancy TG in the relationships between T3 and HDP in non-OWO pregnant women**

| **Outcome** | **Exposure** | **TE** | ***P*** | **PNDE** | ***P*** | **TNIE** | ***P*** | ***P*_adj_** | **Proportion Mediated** |
| --- | --- | --- | --- | --- | --- | --- | --- | --- | --- |
| **HDP** | **FT3** | 1.10 (1.03, 1.17) | 0.008 | 1.07 (1.01, 1.14) | 0.024 | 1.03 (1.02, 1.04) | <0.001 | <0.001 | 27.9% |
|  | **TT3** | 1.18 (1.10, 1.26) | <0.001 | 1.13 (1.06, 1.21) | <0.001 | 1.04 (1.02, 1.06) | <0.001 | <0.001 | 28.1% |
| **PE** | **FT3** | 1.16 (1.04, 1.27) | 0.004 | 1.13 (1.02, 1.24) | 0.028 | 1.02 (1.01, 1.04) | <0.001 | <0.001 | 16.6% |
|  | **TT3** | 1.20 (1.10, 1.33) | <0.001 | 1.15 (1.05, 1.28) | <0.001 | 1.04 (1.01, 1.07) | 0.016 | 0.016 | 23.1% |
| **GH** | **FT3** | 1.06 (0.96, 1.14) | 0.220 | 1.03 (0.94, 1.11) | 0.516 | 1.03 (1.01, 1.04) | <0.001 | <0.001 | 50.1% |
|  | **TT3** | 1.16 (1.06, 1.26) | 0.008 | 1.10 (1.01, 1.21) | 0.032 | 1.05 (1.02, 1.07) | <0.001 | <0.001 | 33.8% |

Effect estimates correspond to a 1-SD increase in FT3 or TT3 (0.94 pmol/L and 0.37 nmol/L, respectively). Mediation analyses were adjusted for age, parity, pre-pregnancy BMI, mode of conception, education level, family history of hypertension, TPOAb positivity, and TgAb positivity.

BMI, body mass index; FT3, free triiodothyronine; GH, gestational hypertension; HDP, hypertensive disorders of pregnancy; OWO, overweight or obesity; PE, preeclampsia; PNDE, pure natural direct effect; SD, standard deviation; TE, total effect; TG, triglyceride; TNIE, total natural indirect effect; TgAb, thyroglobulin antibody; TPOAb, thyroid peroxidase antibody; T3, triiodothyronine; TT3, total triiodothyronine.
