# Supplementary material for: Associations of early-pregnancy triiodothyronine and triglycerides with hypertensive disorders of pregnancy: a retrospective cohort study
Source: Front Endocrinol (Lausanne). 2026 Oct 9;17:1975129. doi: 10.3389/fendo.2026.1975129 (PMC13690663; doi:10.3389/fendo.2026.1975129)
Supplement: Supplementary file 2 [file Table2.doc]

**Table S2 Mediation analysis of early-pregnancy TG in the relationships between T3 and HDP in non-GDM pregnant women**

| **Outcome** | **Exposure** | **TE** | ***P*** | **PNDE** | ***P*** | **TNIE** | ***P*** | ***P*_adj_** | **Proportion Mediated** |
| --- | --- | --- | --- | --- | --- | --- | --- | --- | --- |
| **HDP** | **FT3** | 1.09 (1.02, 1.15) | 0.016 | 1.06 (1.00, 1.13) | 0.064 | 1.02 (1.01, 1.03) | <0.001 | <0.001 | 27.0% |
|  | **TT3** | 1.17 (1.10, 1.26) | <0.001 | 1.13 (1.06, 1.21) | 0.004 | 1.04 (1.02, 1.06) | <0.001 | <0.001 | 24.4% |
| **PE** | **FT3** | 1.17 (1.05, 1.28) | 0.008 | 1.14 (1.02, 1.24) | 0.012 | 1.02 (1.01, 1.03) | <0.001 | <0.001 | 14.9% |
|  | **TT3** | 1.24 (1.12, 1.36) | <0.001 | 1.19 (1.07, 1.32) | <0.001 | 1.04 (1.01, 1.06) | 0.020 | 0.020 | 19.2% |
| **GH** | **FT3** | 1.03 (0.94, 1.11) | 0.472 | 1.01 (0.93, 1.09) | 0.856 | 1.02 (1.01, 1.03) | <0.001 | <0.001 | 75.6% |
|  | **TT3** | 1.13 (1.04, 1.22) | 0.004 | 1.09 (1.00, 1.18) | 0.060 | 1.04 (1.02, 1.06) | 0.004 | 0.008 | 33.0% |

Effect estimates correspond to a 1-SD increase in FT3 or TT3 (0.94 pmol/L and 0.37 nmol/L, respectively). Mediation analyses were adjusted for age, parity, pre-pregnancy BMI, mode of conception, education level, family history of hypertension, TPOAb positivity, and TgAb positivity.

BMI, body mass index; FT3, free triiodothyronine; GDM, gestational diabetes mellitus; GH, gestational hypertension; HDP, hypertensive disorders of pregnancy; PE, preeclampsia; PNDE, pure natural direct effect; SD, standard deviation; TE, total effect; TG, triglyceride; TNIE, total natural indirect effect; TgAb, thyroglobulin antibody; TPOAb, thyroid peroxidase antibody; T3, triiodothyronine; TT3, total triiodothyronine.
