# Supplementary material for: Associations of early-pregnancy triiodothyronine and triglycerides with hypertensive disorders of pregnancy: a retrospective cohort study
Source: Front Endocrinol (Lausanne). 2026 Oct 9;17:1975129. doi: 10.3389/fendo.2026.1975129 (PMC13690663; doi:10.3389/fendo.2026.1975129)
Supplement: Supplementary file 3 [file Table3.doc]

**Table S3 Exploratory mediation analyses of other lipid biomarkers in the associations between T3 and HDP**

| **Outcome** | **Mediator** | **Exposure** | **TE** | ***P*** | **PNDE** | ***P*** | **TNIE** | ***P*** | ***P*_adj_** | **Proportion Mediated** |
| --- | --- | --- | --- | --- | --- | --- | --- | --- | --- | --- |
| **HDP** | **TC** | **FT3** | 1.075 (1.011, 1.136) | 0.032 | 1.072 (1.008, 1.132) | 0.040 | 1.003 (1.000, 1.006) | 0.068 | 0.175 | 4.2% |
|  |  | **TT3** | 1.134 (1.068, 1.198) | <0.001 | 1.131 (1.065, 1.195) | <0.001 | 1.002 (1.000, 1.005) | 0.100 | 0.220 | 2.1% |
|  | **HDL** | **FT3** | 1.075 (1.013, 1.137) | 0.012 | 1.068 (1.005, 1.129) | 0.020 | 1.007 (1.003, 1.012) | <0.001 | <0.001 | 9.9% |
|  |  | **TT3** | 1.134 (1.072, 1.197) | <0.001 | 1.123 (1.061, 1.185) | <0.001 | 1.010 (1.003, 1.018) | 0.004 | 0.072 | 8.3% |
|  | **LDL** | **FT3** | 1.075 (1.013, 1.142) | 0.008 | 1.075 (1.012, 1.141) | 0.008 | 1.000 (0.999, 1.002) | 0.472 | 0.531 | 0.6% |
|  |  | **TT3** | 1.135 (1.075, 1.201) | <0.001 | 1.131 (1.071, 1.197) | <0.001 | 1.004 (1.001, 1.007) | 0.008 | 0.072 | 3.0% |
|  | **Apo A** | **FT3** | 1.075 (1.015, 1.132) | 0.016 | 1.079 (1.018, 1.138) | 0.016 | 0.996 (0.991, 1.001) | 0.084 | 0.202 | -5.4% |
|  |  | **TT3** | 1.134 (1.074, 1.202) | <0.001 | 1.132 (1.072, 1.202) | <0.001 | 1.001 (1.000, 1.004) | 0.200 | 0.343 | 1.0% |
|  | **Apo B** | **FT3** | 1.076 (1.016, 1.147) | 0.008 | 1.067 (1.005, 1.139) | 0.032 | 1.008 (1.001, 1.015) | 0.028 | 0.120 | 11.1% |
|  |  | **TT3** | 1.135 (1.069, 1.198) | <0.001 | 1.128 (1.062, 1.190) | <0.001 | 1.006 (1.000, 1.012) | 0.036 | 0.120 | 5.0% |
|  | **Lp(a)** | **FT3** | 1.075 (1.016, 1.133) | 0.004 | 1.075 (1.016, 1.132) | 0.004 | 1.001 (1.000, 1.002) | 0.324 | 0.449 | 0.8% |
|  |  | **TT3** | 1.133 (1.067, 1.200) | <0.001 | 1.132 (1.066, 1.198) | <0.001 | 1.001 (0.999, 1.003) | 0.240 | 0.393 | 0.8% |
| **PE** | **TC** | **FT3** | 1.145 (1.042, 1.245) | <0.001 | 1.143 (1.042, 1.239) | <0.001 | 1.002 (0.997, 1.007) | 0.464 | 0.531 | 1.4% |
|  |  | **TT3** | 1.169 (1.069, 1.274) | <0.001 | 1.167 (1.069, 1.275) | <0.001 | 1.002 (0.998, 1.006) | 0.468 | 0.531 | 1.1% |
|  | **HDL** | **FT3** | 1.143 (1.045, 1.225) | 0.004 | 1.135 (1.038, 1.218) | 0.008 | 1.007 (1.001, 1.015) | 0.040 | 0.120 | 5.6% |
|  |  | **TT3** | 1.169 (1.072, 1.266) | <0.001 | 1.157 (1.058, 1.254) | <0.001 | 1.010 (1.000, 1.021) | 0.040 | 0.120 | 7.0% |
|  | **LDL** | **FT3** | 1.145 (1.043, 1.245) | <0.001 | 1.145 (1.042, 1.246) | <0.001 | 1.000 (0.999, 1.002) | 0.700 | 0.740 | 0.2% |
|  |  | **TT3** | 1.170 (1.066, 1.264) | <0.001 | 1.167 (1.064, 1.264) | <0.001 | 1.003 (0.999, 1.007) | 0.156 | 0.281 | 1.9% |
|  | **Apo A** | **FT3** | 1.143 (1.039, 1.240) | 0.012 | 1.150 (1.043, 1.248) | 0.004 | 0.994 (0.983, 1.001) | 0.116 | 0.220 | -4.6% |
|  |  | **TT3** | 1.169 (1.071, 1.268) | <0.001 | 1.167 (1.069, 1.263) | <0.001 | 1.002 (0.999, 1.006) | 0.260 | 0.407 | 1.3% |
|  | **Apo B** | **FT3** | 1.145 (1.052, 1.245) | <0.001 | 1.141 (1.050, 1.236) | <0.001 | 1.004 (0.992, 1.016) | 0.376 | 0.501 | 3.1% |
|  |  | **TT3** | 1.170 (1.073, 1.271) | 0.004 | 1.166 (1.073, 1.267) | 0.008 | 1.003 (0.995, 1.011) | 0.464 | 0.531 | 2.3% |
|  | **Lp(a)** | **FT3** | 1.145 (1.042, 1.236) | 0.008 | 1.144 (1.041, 1.236) | 0.008 | 1.001 (0.999, 1.004) | 0.312 | 0.449 | 0.8% |
|  |  | **TT3** | 1.169 (1.070, 1.280) | <0.001 | 1.167 (1.068, 1.279) | <0.001 | 1.002 (1.000, 1.005) | 0.116 | 0.220 | 1.1% |
| **GH** | **TC** | **FT3** | 1.029 (0.959, 1.103) | 0.428 | 1.025 (0.956, 1.098) | 0.500 | 1.004 (1.000, 1.008) | 0.056 | 0.155 | 13.4% |
|  |  | **TT3** | 1.109 (1.034, 1.188) | 0.008 | 1.106 (1.031, 1.183) | 0.008 | 1.003 (0.999, 1.007) | 0.112 | 0.220 | 3.2% |
|  | **HDL** | **FT3** | 1.030 (0.950, 1.108) | 0.404 | 1.023 (0.944, 1.100) | 0.54 | 1.007 (1.002, 1.014) | 0.012 | 0.086 | 23.9% |
|  |  | **TT3** | 1.109 (1.035, 1.190) | 0.008 | 1.098 (1.027, 1.179) | 0.020 | 1.010 (1.002, 1.018) | 0.008 | 0.072 | 10.2% |
|  | **LDL** | **FT3** | 1.030 (0.955, 1.108) | 0.452 | 1.029 (0.955, 1.108) | 0.456 | 1.001 (0.999, 1.003) | 0.516 | 0.563 | 1.9% |
|  |  | **TT3** | 1.110 (1.015, 1.190) | 0.020 | 1.105 (1.012, 1.185) | 0.028 | 1.004 (1.001, 1.009) | 0.016 | 0.096 | 4.2% |
|  | **Apo A** | **FT3** | 1.029 (0.959, 1.107) | 0.368 | 1.032 (0.961, 1.111) | 0.328 | 0.997 (0.990, 1.002) | 0.304 | 0.449 | -9.6% |
|  |  | **TT3** | 1.109 (1.034, 1.188) | 0.012 | 1.108 (1.034, 1.189) | 0.012 | 1.001 (0.999, 1.004) | 0.448 | 0.531 | 0.9% |
|  | **Apo B** | **FT3** | 1.030 (0.947, 1.105) | 0.440 | 1.019 (0.940, 1.092) | 0.636 | 1.011 (1.001, 1.020) | 0.028 | 0.120 | 35.9% |
|  |  | **TT3** | 1.110 (1.029, 1.196) | 0.008 | 1.101 (1.022, 1.189) | 0.016 | 1.008 (1.001, 1.015) | 0.032 | 0.120 | 7.9% |
|  | **Lp(a)** | **FT3** | 1.030 (0.953, 1.120) | 0.408 | 1.029 (0.953, 1.120) | 0.400 | 1.000 (0.999, 1.002) | 0.740 | 0.740 | 0.9% |
|  |  | **TT3** | 1.108 (1.027, 1.184) | 0.012 | 1.108 (1.026, 1.182) | 0.012 | 1.000 (0.998, 1.003) | 0.724 | 0.740 | 0.4% |

Effect estimates correspond to a 1-SD increase in FT3 or TT3 (0.94 pmol/L and 0.37 nmol/L, respectively). Mediation analyses were adjusted for age, parity, pre-pregnancy BMI, mode of conception, education level, family history of hypertension, TPOAb positivity, and TgAb positivity.

Apo A, apolipoprotein A; Apo B, apolipoprotein B; BMI, body mass index; FT3, free triiodothyronine; GH, gestational hypertension; HDL, high-density lipoprotein; HDP, hypertensive disorders of pregnancy; LDL, low-density lipoprotein; Lp(a), lipoprotein(a); PE, preeclampsia; PNDE, pure natural direct effect; SD, standard deviation; TC, total cholesterol; TE, total effect; TG, triglyceride; TNIE, total natural indirect effect; TgAb, thyroglobulin antibody; TPOAb, thyroid peroxidase antibody; T3, triiodothyronine; TT3, total triiodothyronine.
